# Comparative Analysis of Inhibition Efficiency of Chimeric Antisense Oligonucleotides with Various Chemical Modifications and Their Application for Exogenous Control of Gene Expression in *Escherichia coli*

**DOI:** 10.3390/antibiotics15100985

**Published:** 2026-10-06

**Authors:** Antoniya V. Georgieva, Robert Penchovsky

**Affiliations:** Laboratory of Synthetic Biology and Bioinformatics, Faculty of Biology, Sofia University “St. Kliment Ohridski”, 8 Dragan Tzankov Blvd., 1164 Sofia, Bulgaria

**Keywords:** exogenous control of gene expression, antisense oligonucleotides, chemical modifications, *lacZ* gene, logic gates, drug development, gene regulation

## Abstract

**Background:** Antisense oligonucleotides (ASOs) are synthetic nucleic acid analogs that can suppress gene expression through sequence-specific hybridization with target RNA. Enzymatic degradation, low bioavailability, and limited cellular uptake constrain their applications. Chemical modifications to the backbone and sugar moieties can enhance nuclease resistance, improve hybridization affinity, and extend in vivo half-life. This study compared the effects of structural and chemical modifications on the inhibitory activity of cell-penetrating peptide-conjugated ASOs in a bacterial reporter system. **Methods:** Seven ASO variants were evaluated, encompassing nucleotide deletions and additions, locked nucleic acid residues, 2′-O-methyl modifications, thiophosphoramidate morpholino (TMO) chemistry, and phosphorothioate (PS) linkages. We assessed inhibitory activity in Escherichia coli using a lacZ reporter assay. We quantified their effects by measuring residual β-galactosidase activity, providing a functional readout of reporter inhibition across the seven designs. **Results:** The TMO-modified ASO produced the lowest residual reporter activity among the tested designs, corresponding to 97% inhibition at 120 μg/mL. The PS-modified ASO achieved 92% inhibition at the same concentration. Both designs substantially reduced reporter activity under the tested experimental conditions, with the TMO-modified variant showing the greatest observed inhibition. **Conclusions**: Structural and chemical modifications influenced the inhibitory activity of peptide-conjugated ASOs in the E. coli lacZ reporter system under the conditions examined. The results support further evaluation of TMO-containing designs for bacterial reporter-gene suppression. Chemical optimization may help develop ASO-based tools for external control of gene expression, with potential applications in synthetic biology, biotechnology, and metabolic engineering.

## 1. Introduction

Antisense oligonucleotides (ASOs) are short, synthetic, single-stranded nucleic acids, typically 16–30 nucleotides in length, designed to hybridize with complementary target RNAs and modulate gene expression through several mechanisms, including enzymatic RNA degradation or blocking translation [1,2]. One pathway involves RNase H-mediated degradation, in which hybridization of an RNase H-compatible ASO to RNA recruits RNase H, leading to RNA cleavage and subsequent suppression of protein translation [3].

The challenges of ASO-based therapies include susceptibility to nucleolytic degradation, reduced binding affinity, and reduced uptake. Advances in chemical modification are addressing these issues. Backbone modifications, such as phosphorothioate linkages (PS), enhance nuclease resistance, while sugar modifications, including 2′-O-methyl groups or locked nucleic acids (LNA), increase binding affinity and stability [1,4]. Conjugation strategies involving lipids or cell-penetrating peptides (CPPs) enhance cellular uptake and tissue distribution. Together, these modifications enable ASOs to retain their activity in vivo and expand their potential, including systemic treatment of bacterial infections.

Our bioinformatics and computational design methods enhance ASO efficiency [1] by enabling precise ASO design and identifying conserved, functionally essential target sites to minimize off-target effects. To evaluate the functional efficacy of these diverse ASO chemical and structural modifications, we selected Escherichia coli as our model bacterial system. *E. coli* is an ideal and widely established platform for synthetic biology and bacterial genetics. We specifically chose the lacZ gene, which encodes β-galactosidase, as our target reporter. The lacZ reporter system is highly sensitive and robust, offering a colorimetric readout that allows precise quantitative measurement of gene expression levels (in Miller units) under various experimental conditions.

Conventional small-molecule antibiotics are increasingly undermined by widespread multidrug resistance (MDR), creating an urgent need for innovative antibacterial drug development approaches. ASOs offer several advantages in this context. First, their sequence specificity enables the precise targeting of essential RNAs, thereby reducing off-target effects. Second, targeting conserved, functionally important RNA regions may constrain some routes to resistance, although resistance can still arise. Third, ASOs can be rapidly re-engineered to target new sequences as resistance emerges, providing a flexible approach to antibacterial design [5,6].

Several features distinguish ASOs from conventional antibiotics and other RNA-based therapies. Sequence specificity can support selective targeting of bacterial transcripts [5]. Their design flexibility allows sequence-directed targeting of bacterial genes, including those encoding essential metabolic enzymes and virulence factors. Previous work targeted FMN riboswitch-containing mRNAs in methicillin-resistant Staphylococcus aureus [6]. The present study instead examines an engineered lacZ reporter in *E. coli*.

RNAs can be programmed to execute Boolean logic functions, such as YES and NOT gates, allowing the synthetic regulation of gene expression in the presence or absence of defined effectors [7]. Computer programs automate the design processes. This capability extends designer ASOs beyond therapeutic silencing into RNA synthetic biology, where molecular computation can control genetic networks.

ASOs are classified into three generations, each with distinct chemical modifications. These chemical modifications play an essential role in ASO function. However, systematic comparative studies evaluating these distinct chemistries within a unified bacterial system remain limited. In the present study, we evaluated the performance of several chemical and structural modifications applied to ASOs designed to operate within a NOT Boolean logic gate. To do so, we established a *lacZ* reporter system to quantify the efficiency of different ASO variants [8]. This approach enabled comparison of the observed reporter activities associated with PS, 2′-OMe, LNA, and TMO designs. Among these, TMO and PS modifications produced the strongest suppression of *lacZ* expression, with TMO achieving up to 97% inhibition and PS reaching 92% inhibition at 120 μg/mL, showing lower residual activity than the other tested designs. To clearly separate our experimental variables, we note that comparisons among ASO2, ASO3, ASO5, ASO6, and ASO7 reduce confounding by target sequence (2′-OMe, unmodified DNA, 2′-OMe gapmer, TMO, and LNA, respectively), as they target the same region; however, ASO5 includes uracil substitutions, and comparisons at equal mass concentrations do not necessarily represent equal molar exposures. In contrast, comparisons involving ASO1 (which has a one-nucleotide deletion) and ASO4 (which includes a 3′-terminal ACCA extension) are confounded by these structural and sequence-length differences, reflecting combined chemical and structural variation.

## 2. Results

**Figure 1:**
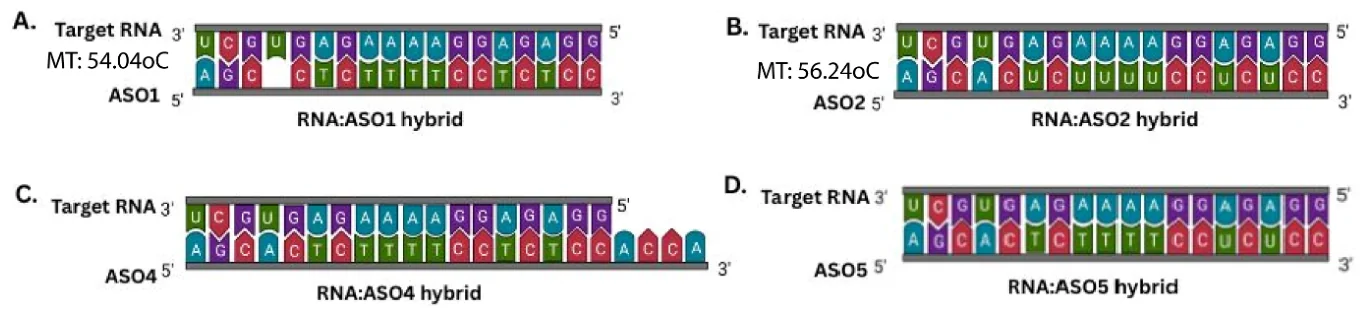
ASO structural modifications. (**A**) Deletion of the adenine at position 4 (ASO1); (**B**) RNA:RNA hybrid (ASO2); (**C**) addition of the ACCA motif at the 3′-terminus (ASO4); (**D**) incorporation of uracil nucleotides near the 3′-terminus (ASO5). Schematics of ASO1, ASO2, ASO4, and ASO5 illustrate the sequence and structural variations. The complete sequences and chemical modifications of all seven ASOs are listed in Table 1.

**Figure 2:**
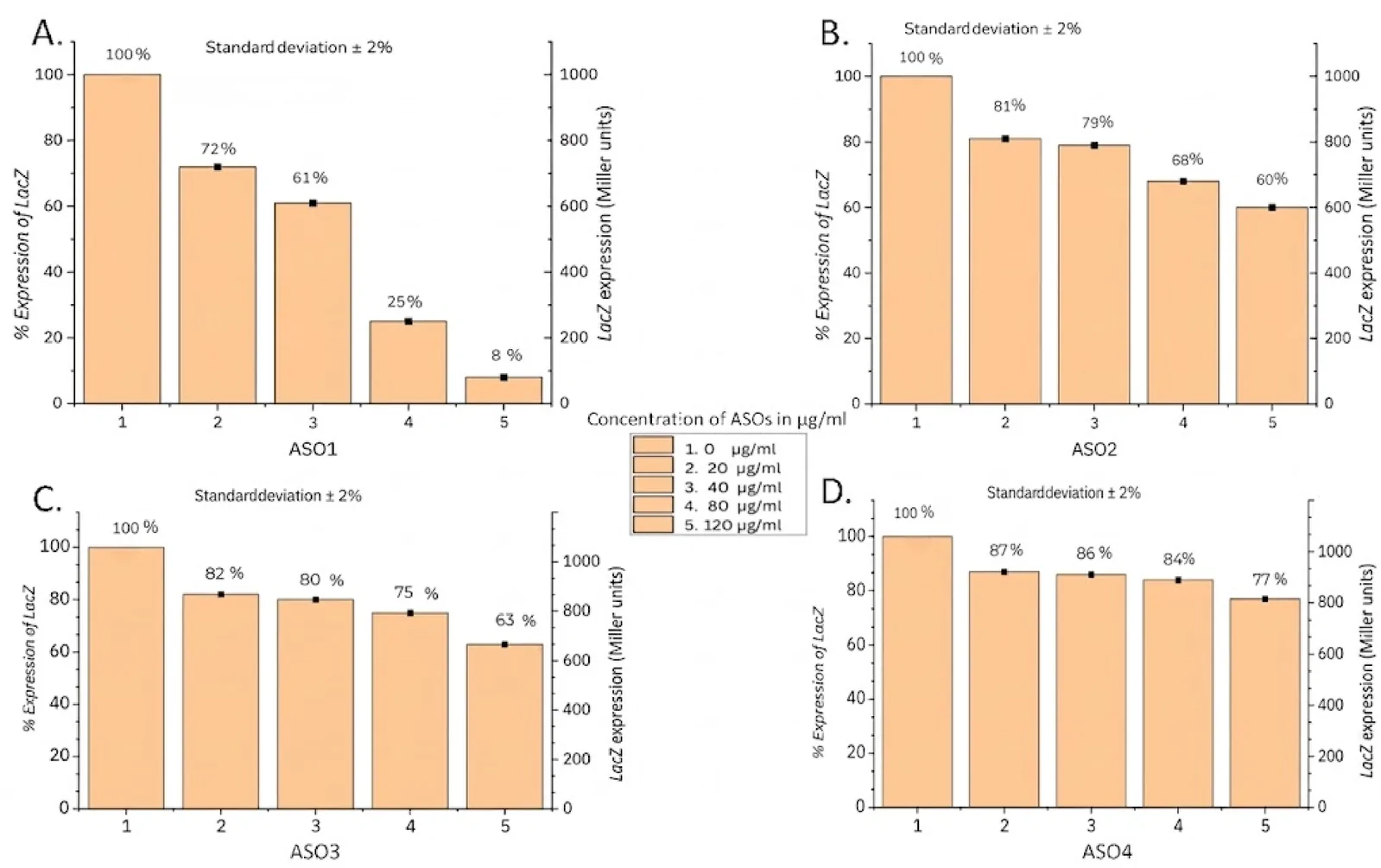
**Inhibition of expression of the *lacZ* gene targeted with four ASO designs.** (**A**) ASO1; (**B**) ASO2; (**C**) ASO3; (**D**) ASO4. Results are presented as mean values from three independent biological replicates, with error bars representing the standard deviation (SD) for each condition. The reported ASO mass concentrations were 0 μg/mL (control), 20 μg/mL, 40 μg/mL, 80 μg/mL, and 120 μg/mL. The resulting values are plotted as percentages of residual activity (left *y*-axis) and in Miller units (right *y*-axis).

**Inhibition of *lacZ* expression with various ASOs:** Our experiments evaluated the inhibitory effects of seven chimeric ASOs with different chemical modifications (Table 1) on *lacZ* gene expression. This assay did not directly measure binding stability. Increasing ASO1 concentration resulted in a consistent inhibitory effect on *lacZ* expression despite the one-nucleotide deletion at position 4 (Figure 1A). Figure 1 displays melting temperatures of 54.04 °C and 56.24 °C for the ASO1 and ASO2 hybrids, respectively, a difference of 2.20 °C. Because these ASOs also differ in chemistry, this comparison does not isolate the deletion effect. PS-modified DNA ASOs can support RNase H-mediated target cleavage, but RNase H dependence and multiple-turnover activity were not measured in this assay. All ASOs were attached to the CPP pVEC. ASO1 is PS-modified (Table 1). A dose-dependent decrease in reporter activity was observed: without ASO1 (control), *lacZ* expression was set to 100%; at 20 μg/mL ASO1, expression was decreased by 28% to 72%; at 40 μg/mL, expression was reduced by 39% to 61%; at 80 μg/mL, expression was decreased by 75% to 25%; and at 120 μg/mL, expression was inhibited by 92% to 8% (Figure 2A). 

ASO2 is modified with 2′-O-methyl groups at every sugar residue (Table 1). It showed a weak inhibitory effect on *lacZ* expression. At 120 μg/mL, expression decreased to 60%, compared with 100% in the control without ASO2 (Figure 2B). Dose-dependent inhibition was observed, with expression decreasing to 81% at 20 μg/mL, 79% at 40 μg/mL, and 68% at 80 μg/mL (Figure 2B). However, the overall effect remained limited; even at the highest concentration tested, residual activity was 60%, corresponding to 40% inhibition. Thus, the observed reduction was smaller than that reported for ASO1 and ASO6. Fully 2′-O-methyl-modified ASOs do not support RNase H-mediated cleavage of their RNA targets and may instead act through steric blocking. The present assay did not establish the mechanism of ASO2 inhibition.

ASO3 weakly inhibited *lacZ* gene expression and was chemically unmodified (Table 1). At 0 μg/mL (control), expression was 100%; at 20 μg/mL, 82%; at 40 μg/mL, 80%; at 80 μg/mL, 75%; and at 120 μg/mL, 63%. Thus, β-galactosidase activity declined modestly in a dose-dependent manner; we did not measure intracellular ASO stability directly (Figure 2C).

ASO4 produced only a minor inhibitory effect on *lacZ* gene expression. At 0 μg/mL (control), expression was 100%; it decreased slightly to 87% at 20 μg/mL, 86% at 40 μg/mL, 84% at 80 μg/mL, and 77% at 120 μg/mL. The plotted results (Figure 2D) showed limited inhibition with the added ACCA motif at the 3′-terminus. ASO4 showed lower efficiency than ASO2 and ASO3, although comparison with ASO2 also involves a difference in chemistry, whereas comparison with ASO3 assesses the added sequence.

**Figure 3:**
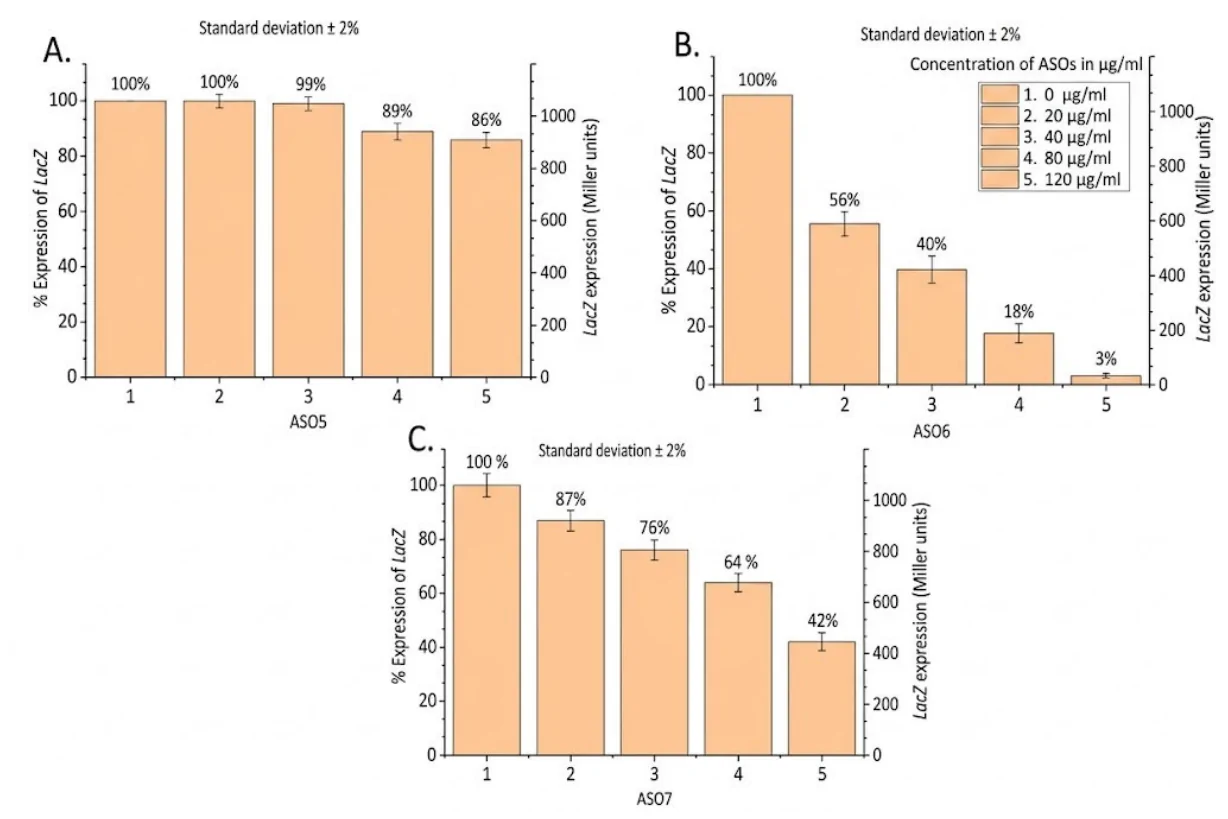
**Inhibition of *lacZ* reporter expression by three modified ASOs.** (**A**) ASO5; (**B**) ASO6; (**C**) ASO7. The reported ASO mass concentrations were 0 μg/mL (control), 20 μg/mL, 40 μg/mL, 80 μg/mL, and 120 μg/mL. Results are presented as means of three independent biological replicates, with error bars representing the standard deviation for each condition. Values are plotted as percentages of residual activity (left *y*-axis) and in Miller units (right *y*-axis).

ASO5 showed the weakest inhibitory activity among all tested variants. At 0 μg/mL (control), *lacZ* expression was set to 100%. Expression remained nearly unchanged at 20 μg/mL (100%) and 40 μg/mL (99%), with only modest decreases to 89% at 80 μg/mL and 86% at 120 μg/mL. The design with 2′-O-methyl-modified termini showed limited inhibition; we did not directly measure stability or hybridization efficiency (Figure 3A).

ASO6 bearing TMO modifications at both termini showed the greatest observed suppression among all tested ASOs at the reported mass concentrations. At 0 μg/mL (control), *lacZ* expression was set to 100%; it decreased sharply to 56% at 20 μg/mL, 40% at 40 μg/mL, 18% at 80 μg/mL, and as low as 3% at 120 μg/mL. This near-complete suppression of *lacZ* expression (Figure 3B) represents the lowest residual reporter activity among the tested designs.

ASO7 produced dose-dependent inhibition of *lacZ* expression. At 0 μg/mL (control), expression was 100%; at 20 μg/mL, 87%; at 40 μg/mL, 76%; at 80 μg/mL, 64%; and at 120 μg/mL, 42%. These results (Figure 3C) showed substantial but incomplete inhibition by ASO7 and a higher residual activity than with ASO6.

Across the seven ASO variants, residual *lacZ* reporter activity at 120 μg/mL was 8% for ASO1, 60% for ASO2, 63% for ASO3, 77% for ASO4, 86% for ASO5, 3% for ASO6, and 42% for ASO7 (Figure 2 and Figure 3; Figure S1). These values correspond to reductions of 92%, 40%, 37%, 23%, 14%, 97%, and 58%, respectively. ASO6 had the lowest observed residual activity, followed by ASO1 and ASO7. The 3% versus 8% residual activities for ASO6 and ASO1 represent a descriptive difference of 5 percentage points; these means alone do not establish a statistically significant difference between the ASOs. In addition, ASO1 differs in sequence and length, so its comparison with ASO6 cannot isolate the contribution of chemical modification.

We performed growth-curve experiments in three independent biological replicates for all seven ASO designs at the reported maximum concentration (120 μg/mL). Comparisons of area under the curve (AUC) using two-tailed Student’s *t*-tests showed no statistically significant difference between each treated group and the untreated control (*p* > 0.05). Figure S2 illustrates the growth curves for ASO6 and the untreated control. A nonsignificant difference does not establish that the growth curves are identical or rule out other forms of toxicity.

## 3. Discussion

Different chemical modifications of the ASO nucleotide backbone, as well as structural changes and CPP attachment, affect its stability and efficiency. To compare reporter inhibition across ASO designs, we tested various chemical modifications and structural changes on a chimeric ASO bearing the CPP pVEC. We examined the effects of nucleotide deletions (ASO1) and additions (ACCA motif at the 3′-terminus of ASO4), fully 2′-O-methyl-modified nucleotides in ASO2 and uracil-containing terminal modifications in ASO5, and various chemical modifications.

ASO1, modified with a PS backbone and with adenine at position 4 removed from its sequence, showed dose-dependent suppression of *lacZ* expression, achieving 92% inhibition at the highest concentration tested (Figure 2A). This result aligns with well-established data showing that PS modifications can enhance ASO nuclease resistance, although they can sometimes increase non-specific interactions [1]. ASO1 showed strong reporter inhibition despite the deletion.

In contrast, ASO2 to ASO5 demonstrated limited inhibition, with different chemical and structural configurations (Figure 2B,D and Figure 3A). ASO2 was designed with 2′-O-methyl modifications at every sugar residue. At 120 μg/mL, residual reporter activity was 60%, corresponding to 40% inhibition. While 2′-O-methyl substitutions improve resistance to nucleases and reduce immunogenicity [9,10], fully 2′-O-methyl-modified ASOs do not support RNase H-mediated target cleavage and can act through steric blocking [11]. This distinction does not, by itself, establish single-turnover kinetics.

ASO3 and ASO4 showed 37% and 23% reductions in reporter activity, respectively, at 120 μg/mL. ASO4 included an ACCA motif intended to promote RNase P-mediated cleavage, but its residual activity was higher than that of ASO3. These observations do not establish the underlying mechanism. We did not directly measure intracellular degradation or RNase P activity, and the contribution of the sequence extension requires a controlled comparison.

ASO5, which combined 2′-O-methyl-modified termini with an unmodified central DNA segment, produced only 14% inhibition at 120 μg/mL. ASO6 produced the greatest observed reporter suppression, achieving 97% inhibition of *lacZ* expression at the highest tested concentration. ASO6 incorporated TMO modifications, which contain morpholino subunits and thiophosphoramidate internucleoside linkages. Our previous studies have shown nuclease resistance and favorable RNA hybridization properties in TMO oligonucleotides and chimeras [12].

The strong reporter suppression observed with ASO6 is compatible with favorable properties of TMO-containing designs reported in the literature [12]. However, our assays did not quantify binding affinity, intracellular stability, cellular uptake, or RNase H cleavage kinetics. RNase H compatibility depends on the complete oligonucleotide architecture, including the DNA segment; TMO modification alone does not establish RNase H recruitment.

ASO7, incorporating LNA modifications, also demonstrated substantial inhibition, though not to the same extent as ASO6. LNAs contain a methylene bridge that locks the ribose ring into a rigid conformation, thereby significantly enhancing the binding affinity and thermal stability of the ASO–RNA duplex. This modification produced moderate to strong inhibitory effects in the lacZ system, although we did not measure stability here. Overall inhibition remained lower than that observed for the TMO gapmer; this study did not measure binding affinity. Nonetheless, LNAs remain valuable candidates because they can form potent, stable duplexes, making ASO7 a candidate for further evaluation [1].

**Figure 4:**
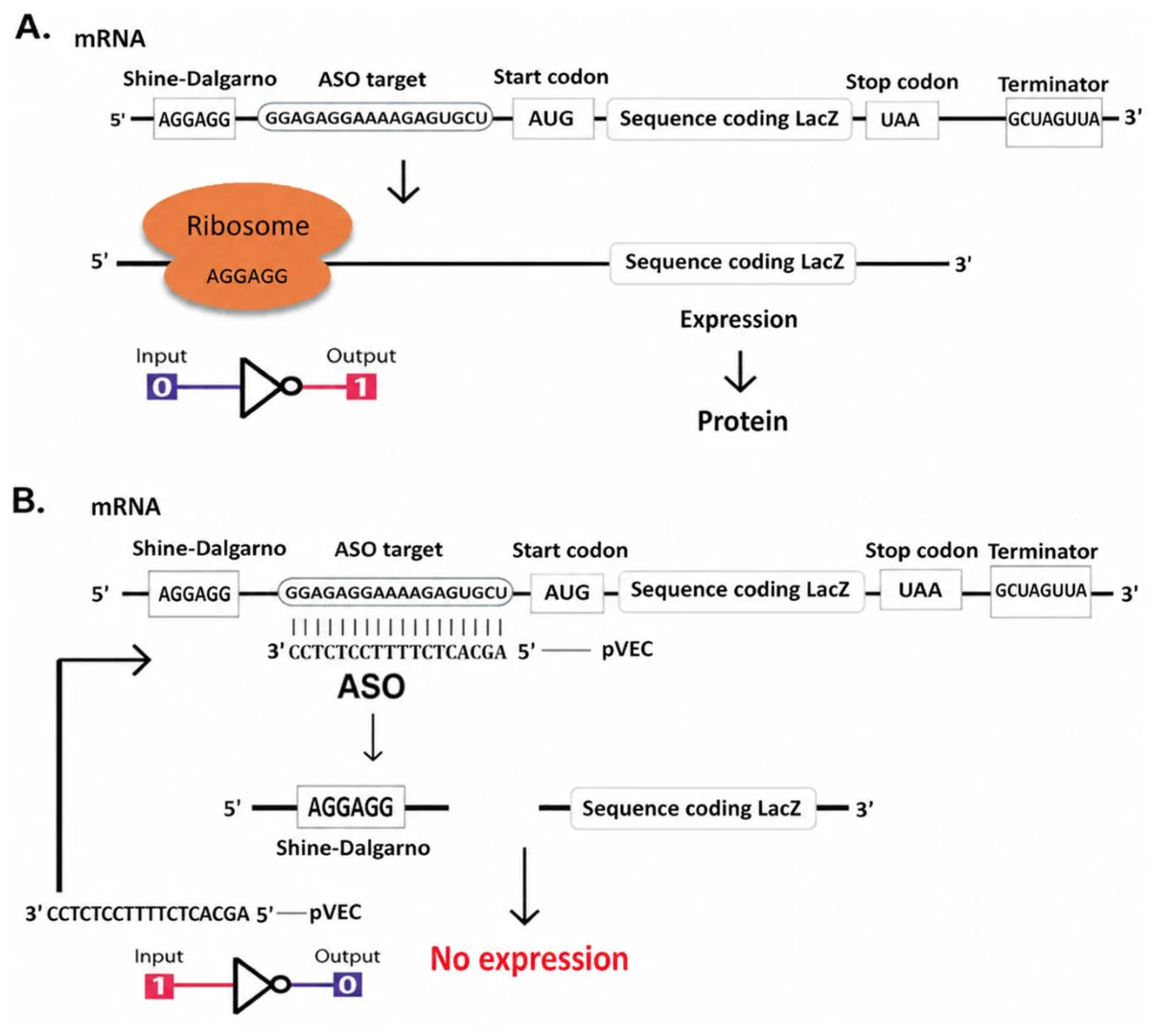
A NOT Boolean logic gate strategy for synthetic control of gene expression based on inhibition of mRNA expression by ASOs in *E. coli*. (**A**) Basic structural elements of a reporter system for quantifying lacZ expression. Without the ASO (input = 0), the mRNA is translated into LacZ protein (output = 1). (**B**) In the presence of the ASO (input = 1), ASO hybridization inhibits reporter expression (output = 0). RNase H-mediated target cleavage is a proposed mechanism for RNase H-compatible designs; this study did not directly demonstrate it.

These findings demonstrate that TMO gapmer configurations are highly effective in our reporter-gene model. While our previous reports on TMO gapmers show that TMO-modified ASOs can inhibit Staphylococcus aureus growth at lower concentrations than certain other designs with PS modifications of the core [2], further systematic studies are needed to determine whether this efficacy translates to essential gene silencing and antibacterial therapeutics. The current results primarily establish differences in reporter-gene suppression in *E. coli*, providing a valuable foundation for rationally designing modified antisense oligonucleotides [1,13]. The engineered expression system (Figure 4) can also control exogenous gene expression, as the *lacZ* gene can be replaced with another gene of interest.

## 4. Materials and Methods

### 4.1. Bioinformatics Analysis

We conducted various bioinformatics analyses to design the ASOs. The workflow included selecting target RNA sequences from databases such as GenBank (NCBI) [14], ENA [15], and Rfam [16] to identify accessible, single-stranded target regions for ASO hybridization. We also used these databases to gather information on the target mRNA sequence, followed by structural analysis, sequence alignment, and similarity screening against the *E. coli* genome using the NCBI Nucleotide BLAST tool v. 2.17 [17,18] to screen for potential off-target complementarity. We designed the ASO sequence to hybridize to the engineered lacZ reporter mRNA while minimizing complementarity to other *E. coli* transcripts.

We evaluated the complementarity of the selected sequences and the thermodynamic properties of the ASOs using the RNAfold and RNAcofold applets on the ViennaRNA web server [19]. This approach enables the rational design of optimized ASOs. We used these analyses to assess potential self-complementarity and secondary structure; however, computational predictions alone do not establish intracellular hybridization efficiency.

### 4.2. ASO Modifications

To assess ASO efficiency, we applied a series of structural and chemical modifications, encompassing first-, second-, and third-generation designs, to the designed ASOs to evaluate their performance. The first chemical modification introduced into the ASO was PS modification combined with a one-nucleotide deletion. The second modification involved generating an ASO with 2′-O-methyl-modified nucleotides to improve stability. The third design was an unmodified ASO sequence. The fourth variant incorporated an ACCA motif at the 3′-terminus, which was intended to support RNase P-mediated cleavage. We did not directly test the effects of this extension on RNase P activity, hybridization, or stability. The fifth ASO combined 2′-O-methyl-modified nucleotides at both termini with a central DNA segment to enable RNase H activation [20] and used uracil nucleotides near the 3′-terminus (Figure 1). The sixth design employed thiophosphoramidate morpholino (TMO) modifications, which combine morpholino subunits with thiophosphoramidate internucleoside linkages [21]. The final modification involved LNAs, in which a methylene bridge locks the ribose conformation [1], significantly enhancing binding affinity, hybrid stability, and thermal stability, and broadening the therapeutic and diagnostic potential of ASOs.

In addition, the pVEC cell-penetrating peptide (CPP) bearing the amino acid sequence “LLIILRRRIRKQAHAHSK” was covalently conjugated to all seven ASO designs to improve cellular uptake and permeability, thereby creating chimeric ASOs (Table 1) [22,23,24].

All seven chimeric ASOs (Table 1) were purchased from Gene Link Inc. (Elmsford, NY, USA). The company produced the nucleic acid portion of the ASOs by chemical synthesis, using a 5′-amino-modified spacer. All ASOs were purified by high-performance liquid chromatography (HPLC). After that, the C-terminus of the pVEC oligopeptide was attached to the 5′-terminus amino-modified spacer of the ASOs via the zero-length crosslinker 1-ethyl-3-(3-dimethylaminopropyl)carbodiimide hydrochloride (EDC). Next, Gene Link Inc. purified all pVEC-ASO conjugates by denaturing polyacrylamide gel electrophoresis (PAGE) and provided us with the gel image. The company quantified each conjugate and delivered it to us as a dry powder.

### 4.3. ASO lacZ Inhibition Assays in E. coli

The ASO-based system, developed for the exogenous control of gene expression in *E. coli*, was designed to inhibit *lacZ* gene expression by targeting *lacZ* mRNA with ASOs. The reference ASO sequence was 5′-AGCACTCTTTTCCTCTCC-3′; Table 1 (Figure 4) lists the sequence variants and chemical modifications.

We designed a *lacZ* reporter gene-based system to quantify the efficiency of various ASO chemical modifications [8]. This system enables indirect measurement of target gene expression by monitoring β-galactosidase enzymatic activity, one of the most sensitive assays available for tracking ASO-mediated inhibition of gene expression [25]. To evaluate the designer ASOs, we used seven ASO designs with different sequence and chemical features. After plasmid assembly, we introduced the construct into *E. coli.* We selected transformed cells on ampicillin-containing agar plates and transferred the resulting resistant colonies to liquid culture. We grew bacteria to the early exponential phase (OD600 of approximately 0.2 to 0.3) before exposing them to the various ASO formulations.

We performed β-galactosidase assays to quantify *lacZ* expression levels in *E. coli*. We incubated the cells at 37 °C with shaking in LB medium containing ampicillin (100 mg/L) overnight. A permeabilization solution containing 100 mM Na_2_HPO_4_, 20 mM KCl, 2 mM MgSO_4_, 0.8 mg/mL hexadecyltrimethylammonium bromide, 0.4 mg/mL sodium deoxycholate, and 5.4 μL/mL β-mercaptoethanol was applied. We added 20 μL of LB culture to 80 μL of the permeabilization solution and mixed well. We measured cell culture absorbance at 600 nm (Abs 600). We transferred the mixture to tubes and added 600 μL of substrate solution containing o-nitrophenyl-β-galactoside (ONPG) and recorded the time of addition. After sufficient color had developed, we added 700 μL of a stop solution containing 1 M sodium carbonate (Na_2_CO_3_). Next, we transferred the tubes to a microfuge and centrifuged them at 5000 rpm for 5 min. We transferred the supernatant to cuvettes and measured absorbance at 420 nm (Abs 420). Enzyme activity was calculated in Miller units (MU) using the formula: MU = 1000 × Abs 420/(Abs 600 × V × T), where T is the reaction time in minutes, and V is the volume of culture used in the assay in mL.

We conducted the β-galactosidase assays in three independent biological replicates (each started from separate bacterial colonies on different days) and calculated average values and standard deviations across these trials. For quantitative comparisons, we normalized enzymatic activity relative to the untreated control group (0 μg/mL ASO), defined as 100% *lacZ* expression (baseline Miller units). To define silencing efficacy, we set objective thresholds: ‘substantial inhibition’ was defined as >50% reduction in *lacZ* expression (corresponding to <50% residual activity), and ‘limited inhibition’ as <50% reduction in expression. Statistical analysis was performed using a one-way analysis of variance (ANOVA) followed by Dunnett’s post hoc test to compare each ASO concentration effect against the untreated control. The reported significance thresholds were *p* < 0.05 and *p* < 0.01. Comparisons of ASO-treated groups with the untreated control do not by themselves test differences between ASOs.

## 5. Conclusions

This study provides a comparative evaluation of multiple chemical and structural modifications among seven ASO designs, demonstrating their potential for synthetic control of bacterial gene expression. The methodological framework established here offers a potential strategy for targeted gene knockdown in *E. coli* and could serve as a valuable reference for optimizing chemical modifications in bacterial gene control. While the pVEC cell-penetrating peptide has been shown to assist transport across various cell types, further studies are needed to determine whether these findings extend to eukaryotic systems or broader antibacterial therapeutics. Nonetheless, this comparative study underscores the utility of ASO design variations for the synthetic control of gene expression.

In this *E. coli lacZ* reporter system, ASO6 with TMO-modified termini produced the lowest observed residual reporter activity at the highest tested mass concentration. The results support further investigation of modified ASOs for synthetic control of bacterial gene expression. Statistical comparisons between ASOs and controls must be distinguished from direct comparisons between ASOs, and the effects of chemistry must be interpreted in light of differences in sequence, length, and molar exposure. Further validation is needed before extending these findings to other organisms, essential gene targets, or antibacterial applications.

All experiments adhered to the good laboratory practice guidelines and current bioethical considerations.

## Figures and Tables

**Table 1:** Designed ASO sequences and their modifications. Cell-penetrating peptide [p-VEC], thiophosphoramidate morpholino [TMO], disulfide thiol [SS-C6], spacer C12 [Sp-C12], 2′-O-methyl [m], phosphorothioate [PS], and LNA (+) modifications are indicated.

Name	Sequence (5′–3′)	Length (nt)
ASO1	[p-VEC][SS-C6][Sp-C12]A[PS]G[PS]C[PS]C[PS]T[PS]C[PS]T[PS]T[PS]T[PS]T[PS]C[PS]C[PS]T[PS]C[PS]T[PS]C[PS]C	17
ASO2	[p-VEC][SS-C6][Sp-C12][mA][mG][mC][mA][mC][mT][mC] [mT][mT][mT][mT][mC][mC][mT][mC][mT][mC][mC]	18
ASO3	[p-VEC][SS-C6][Sp-C12]AGCACTCTTTTCCTCTCC	18
ASO4	[p-VEC][SS-C6][Sp-C12]AGCACTCTTTTCCTCTCCACCA	22
ASO5	[p-VEC][SS-C6][Sp-C12][mA][mG][mC][mA]CTCTTTTCC [mU][mC][mU][mC][mC]	18
ASO6	[p-VEC][SS-C6][Sp-C12][TMO-A][TMO-G][TMO-C][TMO-A] CTCTTTTCC[TMO-T][TMO-C][TMO-T][TMO-C][TMO-C]	18
ASO7	[p-VEC][SS-C6][Sp-C12][+A][+G][+C][+A][+C][+T][+C] [+T][+T][+T][+T][+C][+C][+T][+C][+T][+C][+C]	18

## Data Availability

All data generated in this study are presented in this manuscript.

## Supplementary Materials

The following supporting information can be downloaded at: https://www.mdpi.com/article/10.3390/antibiotics15100985/s1. Figures S1 and S2. Figure S1. Comparative analysis of LacZ gene expression against the seven ASOs at concentrations of A. 20; B. 40; C. 80; and D.120 μg/mL. ASO1 and ASO6 showed the highest LacZ gene suppression. At 120 μg/mL, ASO1 and ASO6 yielded 8% and 3%, respectively, indicating that these modifications are the most effective. Figure S2. The growth curve of E. coli with 60 µM ASO6 (solid line in red) and without ASO6 (dashed line in black).
