# Supplementary material for: Comparative Analysis of Inhibition Efficiency of Chimeric Antisense Oligonucleotides with Various Chemical Modifications and Their Application for Exogenous Control of Gene Expression in Escherichia coli
Source: Antibiotics (Basel). 2026 Oct 6;15(10):985. doi: 10.3390/antibiotics15100985 (PMC13700686; doi:10.3390/antibiotics15100985)
Supplement: Supplementary file 1 [file antibiotics-15-00985-s001.zip › antibiotics-4485463-supplementary.pdf]

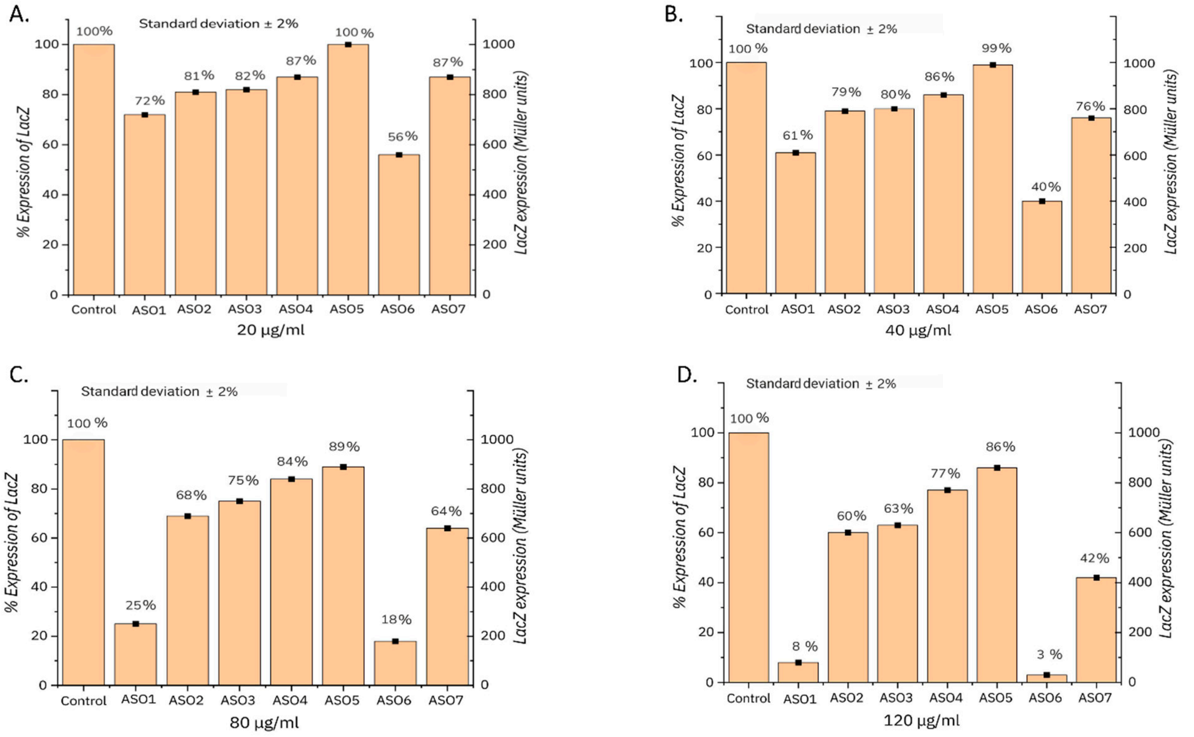

Figure S1. **Comparative analysis of LacZ gene expression against the seven ASOs at concentrations of** A. 20; B. 40; C. 80; and D.120 µg/mL. ASO1 and ASO6 showed the highest LacZ gene suppression. At 120 µg/mL, ASO1 and ASO6 yielded 8% and 3%, respectively, indicating that these modifications are the most effective.

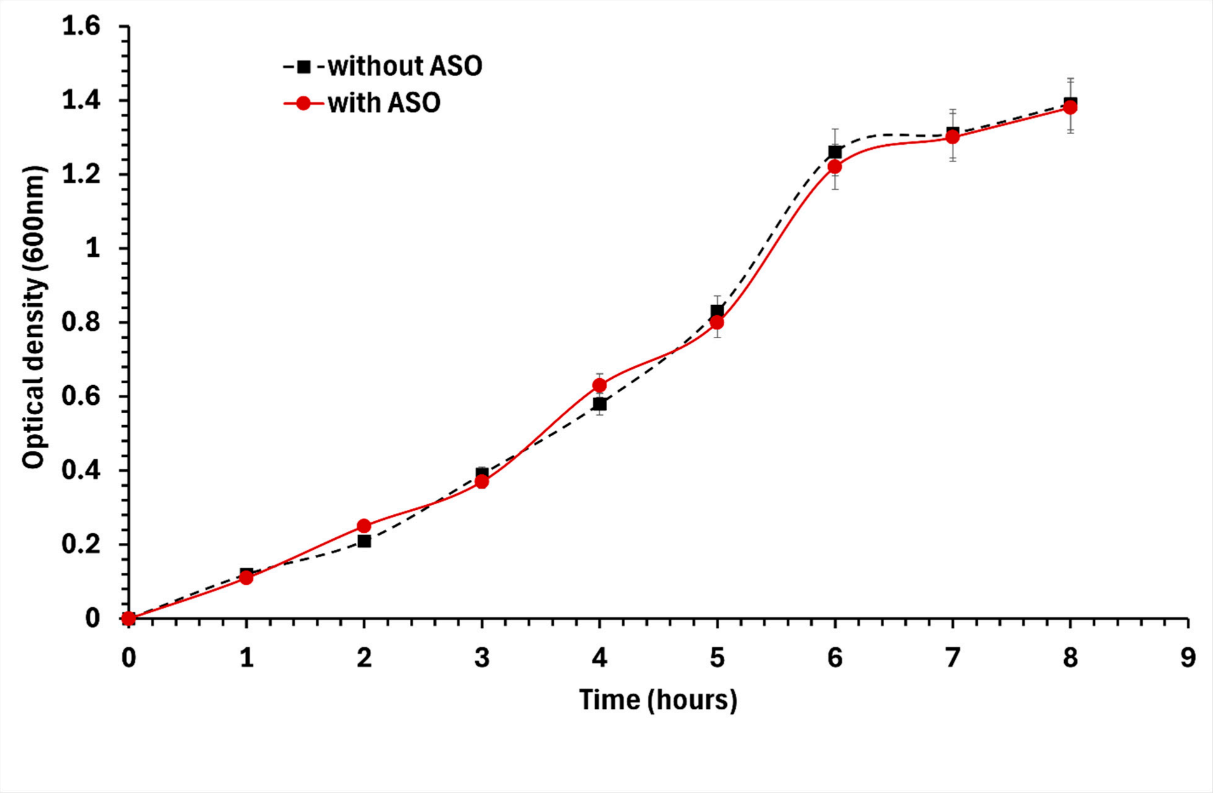

Figure S2. **The growth curve of *E. coli* with 60 μM ASO6 (solid line in red) and without ASO6 (dashed line in black).**
